# “Protein aggregates” contain RNA and DNA, entrapped by misfolded proteins but largely rescued by slowing translational elongation

**DOI:** 10.1111/acel.13326

**Published:** 2021-03-31

**Authors:** Robert J. Shmookler Reis, Ramani Atluri, Meenakshisundaram Balasubramaniam, Jay Johnson, Akshatha Ganne, Srinivas Ayyadevara

**Affiliations:** ^1^ Central Arkansas Veterans Healthcare System Little Rock AR USA; ^2^ Department of Geriatrics University of Arkansas for Medical Sciences Little Rock AR USA; ^3^ BioInformatics Program University of Arkansas for Medical Sciences and University of Arkansas at Little Rock Little Rock AR USA

**Keywords:** aggregation, Alzheimer’s disease, apolipoprotein E, beta amyloid, cotranslational misfolding, DNA, endogenous viruses, functional annotation, gene ontology, neurodegeneration, nucleic acid sequence, nucleic acids, protein aggregates, proteomics, retrotransposons, RNA

## Abstract

All neurodegenerative diseases feature aggregates, which usually contain disease‐specific diagnostic proteins; non‐protein constituents, however, have rarely been explored. Aggregates from SY5Y‐APP_Sw_ neuroblastoma, a cell model of familial Alzheimer's disease, were crosslinked and sequences of linked peptides identified. We constructed a normalized “contactome” comprising 11 subnetworks, centered on 24 high‐connectivity hubs. Remarkably, all 24 are nucleic acid‐binding proteins. This led us to isolate and sequence RNA and DNA from Alzheimer's and control aggregates. RNA fragments were mapped to the human genome by RNA‐seq and DNA by ChIP‐seq. Nearly all aggregate RNA sequences mapped to specific genes, whereas DNA fragments were predominantly intergenic. These nucleic acid mappings are all significantly nonrandom, making an artifactual origin extremely unlikely. RNA (mostly cytoplasmic) exceeded DNA (chiefly nuclear) by twofold to fivefold. RNA fragments recovered from AD tissue were ~1.5‐to 2.5‐fold more abundant than those recovered from control tissue, similar to the increase in protein. Aggregate abundances of specific RNA sequences were strikingly differential between cultured SY5Y‐APP_Sw_ glioblastoma cells expressing *APOE3* vs. *APOE4*, consistent with APOE4 competition for E‐box/CLEAR motifs. We identified many G‐quadruplex and viral sequences within RNA and DNA of aggregates, suggesting that sequestration of viral genomes may have driven the evolution of disordered nucleic acid‐binding proteins. After RNA‐interference knockdown of the translational‐procession factor EEF2 to suppress translation in SY5Y‐APP_Sw_ cells, the RNA content of aggregates declined by >90%, while reducing protein content by only 30% and altering DNA content by ≤10%. This implies that cotranslational misfolding of nascent proteins may ensnare polysomes into aggregates, accounting for most of their RNA content.

## Graphical abstract

Nucleic acid‐binding proteins are shown attaching to RNA and DNA, and then undergoing oxidation, thermal denaturation (reversible or irreversible), or other PTMs that destabilize their secondary structure sufficiently to allow aggregation to occur, an essentially irreversible process. Nascent polypeptides as they are synthesized by ribosomes provide another source of transiently misfolded proteins that can aggregate without oxidation or other trigger for denaturation. The sequence shown is not obligatory, in that disordered proteins could also bind nucleic acids *after* aggregating.

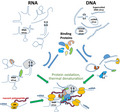

**Abbreviations**
AD: Alzheimer's disease
AMC: age‐matched controls
APOE: *APOE*, apolipoprotein E (protein, gene)
Aβ_1–42_: Amyloid beta (residues 1–42)
ChIP‐seq: Chromatin ImmunoPrecipitation ‐ sequencing
CLEAR: Coordinated Lysosomal Expression and Regulation, a binding motif
DNA: deoxyribonucleic acid
EEF2: *EEF2*, eukaryotic elongation factor 2 (protein, gene)
G4BP: G‐quadruplex binding protein
MAP1A: microtubule‐associated protein 1A
NA: nucleic acids
NS: not significant (referring to a difference between groups)
OE: overexpressing
PTM: post‐translational modification
RNA: ribonucleic acid
TF: transcription factor

## 1 INTRODUCTION

Protein aggregation increases inexorably with aging in all animal species and in all tissues examined (Ayyadevara, Balasubramaniam, Johnson, et al., 2016; Ayyadevara, Balasubramaniam, Suri, et al., 2016; Brignull et al., 2007; Cohen et al., 2009; David et al., 2010; Dillin & Cohen, 2011; Reis‐Rodrigues et al., 2012). Specific aggregate components, diagnostic for each human neurodegenerative disease, are thought to play causal roles because their pathology‐associated mutation and/or overexpression are sufficient to confer heritable neuropathy in human pedigrees and in transgenic‐animal models (Bandyopadhyay et al., 2007; Dillin & Cohen, 2011; Li et al., 2013; Miller et al., 2010; Roodveldt et al., 2009). Proteins that require structural flexibility often incorporate disordered regions, thus rendering them vulnerable to aggregation; other proteins are only susceptible to aggregation after oxidation or specific post‐translational modifications (Ayyadevara et al., 2015, 2017; Ayyadevara, Balasubramaniam, Parcon, et al., 2016).

We recently developed improved click‐chemistry crosslinking reagents and analytical software to identify adjacent proteins in aggregates, based on peptide‐peptide crosslinking, and we applied it to define the protein‐adherence network, or “contactome”, of aggregates. We began with total, sarkosyl‐insoluble aggregates isolated from SY5Y‐APP_Sw_ human neuroblastoma cells (Ayyadevara et al., 2017), a model of familial Alzheimer's disease (fAD). This work revealed a complex, non‐random structure of aggregates in which megahubs (very‐high‐connectivity hubs with ≥100 partners) and hub connectors (low‐connectivity proteins linking large hubs) contribute functionally to the assembly of large aggregates (Balasubramaniam et al., 2019). We noted marked enrichment among megahubs for large structural proteins such as titin, ankyrins 1 – 3, nesprins 1 – 3, MAP1A, and other neurofilament proteins, purely as a consequence of their size. We also observed significant enrichment for a variety of nucleic acid‐binding proteins (Balasubramaniam et al., 2019).

## 2 RESULTS

### 2.1 The aggregate interactome

**FIGURE 1:**
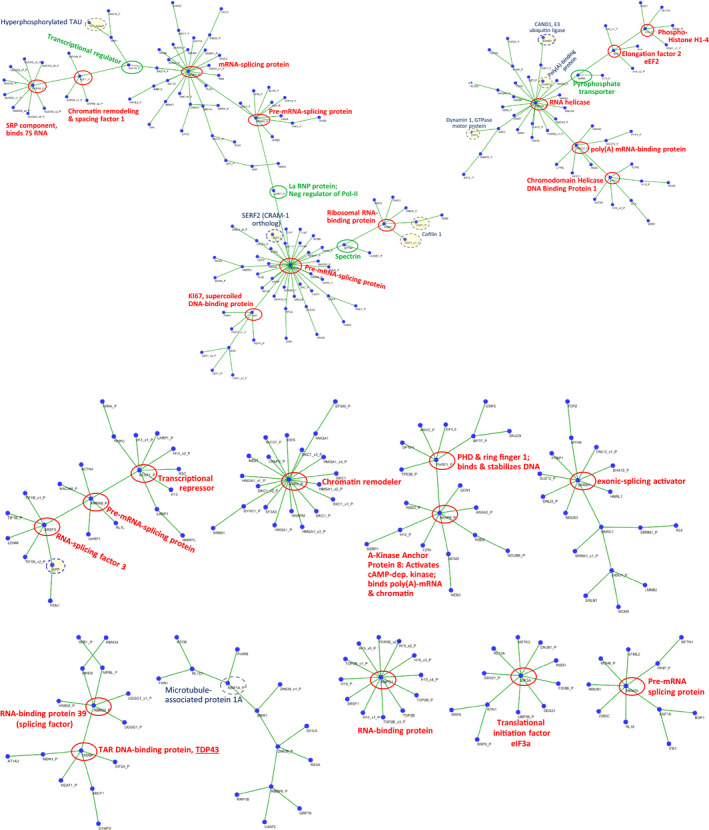
The “aggregate contactome” of proteins isolated from SY5Y‐APP_Sw_ human neuroblastoma cells, an in vitro model of familial AD. The contactome was generated from proteomic data for cross‐linked peptide pairs in sarkosyl‐insoluble aggregates, using a modified version of X‐link Identifier (Balasubramaniam et al., 2019; Du et al., 2011), requiring ≥10 spectral hits per protein observed in at least 2 of 3 replicate crosslinking experiments. Hits were normalized to hub length (amino acids in the most abundant isoform). Red circles highlight central hubs with 5 or more large‐hub interactors; green circles show smaller hub‐connectors, which join major hubs not otherwise connected. Other proteins of interest are indicated by dashed gray circles

To compensate for protein size variation, we reassessed the aggregate interactome with normalization for protein length. The intra‐aggregate contactome, based on length‐normalized connectivity (interaction number per residue), fell into 11 clusters comprising 24 “central hubs” (Figure 1; hubs with >4 edges, indicated by red circles). Four “hub connectors” of low degree (≤4 edges; green circles) bring together large hubs not otherwise connected. Remarkably, all 24 central hubs and 2 of 4 hub connectors are nucleic acid‐binding proteins (Figure 1), revealing a striking enrichment for proteins that bind RNA (*N* = 16; *p *< 3E‒150), or bind DNA (*N* = 6; *p *< 2E‒20), or both (*N* = 2). This supports and extends our earlier observation that nucleic acid‐binding proteins are especially susceptible to aggregation (Balasubramaniam et al., 2019) and led us to inquire whether their targets, RNA and DNA, might also be present in the entities we call “protein aggregates”.

### 2.2 Quantitation of aggregate nucleic acids from AD *vs*. control hippocampus, or human glioma cells

**FIGURE 2:**
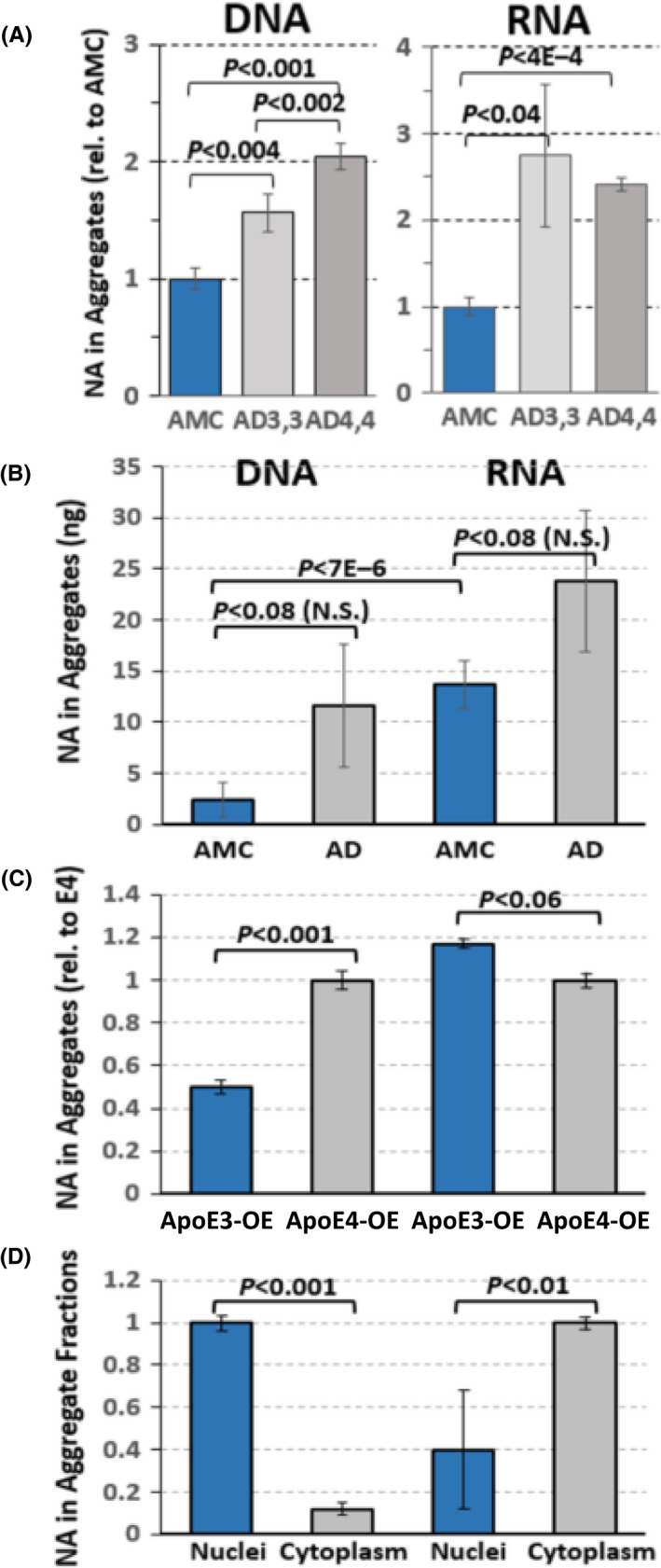
Recovery of DNA and RNA from aggregates. DNA and RNA were extracted and quantified from insoluble aggregates, isolated from hippocampi of AMC (APOE ε3/ε3), or AD (ε3/ε3 or ε4/ε4) individuals (A, each *N* = 3); or from similar mixes of APOE ε3/ε3 and ε3/ε4 individuals (B, each *N* = 5–6). (C, D) DNA and RNA were extracted from insoluble aggregates from T98G glioma cells. C, independent cultures of T98G cells overexpressing APOE ε3 from a transgene were compared to cultures expressing ε4. D, T98G cells without any transgene were lysed in 0.5% NP40, and nuclei separated from cytoplasm. DNA was chiefly associated with nuclear aggregates, and RNA with cytoplasmic aggregates, as expected. Means ±SDs are shown. *p* values reflect 2‐tailed heteroscedastic t tests.

We isolated total sarkosyl‐insoluble aggregates from hippocampal tissue of individuals diagnosed with Alzheimer's disease (AD) and confirmed by histopathological markers, and from age‐matched controls (AMC) without dementia or AD‐diagnostic markers (amyloid deposits or hyperphosphorylated tau). From equal initial weights of hippocampus, quantified recoveries of nucleic acids increased in AD aggregates, over those in controls, by 1.5‐ to 2‐fold for DNA, and ~twofold for RNA (Figure 2A,B). These elevations did not differ significantly from the difference in protein content of total aggregates, which was ~60% higher in AD than in controls, in close agreement with previous results (Ayyadevara, Balasubramaniam, Parcon, et al., 2016). Among normal controls, there was fourfold to sixfold more RNA than DNA in total sarkosyl‐insoluble aggregates (*p *< 1E–5), regardless of the methods used for separation and quantitation (see Experimental Procedures). For AD samples, nucleic acid recoveries were higher and more variable, with roughly twice as much RNA as DNA (Figure 2B).

Apolipoprotein E (*APOE*) gene alleles are the leading genetic risk factors for AD, with at least fourfold increased AD risk for each *APOE* ε4 allele (abbreviated *APOE4, ε4,* or *E4*), and increased severity of aggregate‐associated neuropathology for AD carriers of *APOE4* alleles (Neu et al., 2017; Parker et al., 2005). We recently reported that the concerted transcription of autophagy genes is disrupted in the human glioblastoma cell line T98G, when overexpressing an *APOE4* transgene rather than *APOE3* (Parcon et al., 2018). To assess whether the greater nucleic acid content of aggregates in AD *vs*. AMC hippocampi may reflect the disruption of autophagy in AD, we separately analyzed aggregates from T98G cells that overexpress *APOE* ε3 or ε4 from transgenes. The DNA content of T98G/*E4* aggregates was about twice that of T98G/*E3* aggregates (Figure 2C; *p* < 0.001), whereas their RNA content declined a little (<15%, N. S.) with the *APOE4* allele overexpressed.

Most neuropathic aggregates are cytoplasmic, but may also be nuclear or extracellular. When we separated nuclei from cytoplasm of T98G cells prior to aggregate isolation, similar amounts of aggregate protein were recovered from each fraction. However, nuclear aggregates contained mostly DNA and only 40% as much RNA, while cytoplasmic aggregates contained ~10‐fold more RNA than DNA (Figure 2D).

### 2.3 Sequencing data for aggregate nucleic acids

To assess whether DNA and RNA fragments in aggregates are a random sampling from the genome and transcriptome, respectively, these nucleic acids were separately extracted from pooled aggregate preparations from either AD or age‐matched control (AMC) individuals (*APOE* ε3/ε4 heterozygotes; 3 subjects per group), and their sequences determined (UT Southwestern Genomics Core, Dallas TX). DNA fragments were analyzed using a ChIP‐seq protocol, suited to detection of site specificity, and were then mapped to the human genome. RNA fragments underwent a dual screening, comprising a test of peak significance (similar to ChIP‐seq) followed by RNA‐seq analysis of differential abundance, and mapping to the human genome.

Of the 38 loci that showed significant DNA read peaks in at least one pool (each *p* < 10^‒5^ that the reads actually came from a uniform distribution), 25 reached that threshold in AD vs. 28 in AMC tissue, and 26 in T98G cells carrying *APOE3* vs. 30 in *APOE4* cells (Table 1, Supplementary Table S1). Each group contains 17‒20 peak loci with *p* < 10^‒8^, and one locus with *p* < 10^‒50^. Considering that a random representation of DNA reads would display a flat (uniform) distribution, these data provide decisive evidence that aggregate DNA fragments are not random, but most likely reflect specific binding sites for proteins that ensnared them into aggregates. Only 5 of these 38 DNA peaks (13%) mapped to known genes or ORFs: RP5‐857K21.4, LINC00486, DUX4L26, MAMDC2‐AS1, and ROCK1P1. The other 33 peaks mapped to *intergenic regions*. Of the 24 chromosomes represented, Y had the most DNA reads (47‒98 reads in each of 6 peaks), followed by chromosomes 4 (3 peaks of 48‒53 reads), 1 (2 peaks of 57‒65 reads), 10 (2 peaks of 35‒45 reads), 16 (2 peaks of 26‒35 reads), 17 (2 peaks of 24‒37 reads), and 21 (4 peaks of 9‒17 reads). Neither the numbers of DNA peaks or reads differed significantly between the groups compared (AD vs. AMC, or E3 vs. E4), with the sole exception of a Y‐chromosome peak mapping at 26,638,004‒ 26,638,595, which yielded only 5 reads in aggregates from AD, but 59 for AMC (Chi^2^ *p* < 0.005) and a range of 52‒63 reads for the 3 non‐AD samples.

**TABLE 1:** DNA Fragments Recovered from AD, AMC Aggregates (ChIP‐seq analysis)

Chrom.	Region	Length	5’ Gene	5’ distance	3’ Gene	3’ Distance	AD Peaks	AMC Peaks	AD Peak *p*‐value	AMC Peak *p*‐value	Gene Description (Nearest Gene)
**1**	633735..634300	566	RP5‐857 K21.3	36236	**RP5‐857 K21.4**	0	1	4	4. E−02	4. E−06	Transcribed processed pseudogene; also in all aggregate RNAs
**1**	143202361..143202903	543	RP11‐344P13.1	21621836	CH17‐333 M13.2	140276	56	51	3. E−29	9. E−31	
**2**	32916116..32916684	569	**LINC00486**	0	AL121656.5	10442	2	2	4. E−11	2. E−13	Long Intergenic Non‐Protein‐Coding RNA 486; also in all aggregate RNAs
**2**	89830806..89831378	573	5S_rRNA	230125	IGKV2D−40	20412	11	11	2. E−05	9. E−06	
**3**	75668953..75669543	591	**DUX4L26**	0	LINC00960	2847	1	1	4. E−11	4. E−11	Double Homeobox 4‐Like 26
**3**	93470298..93470861	564	HSPE1P19	3208589	RNU6‐488P	372902	2	1	6. E−57	1. E−63	
**4**	49149228..49149839	612	TPI1P4	132073	AC118282.3	47363	34	27	9. E−11	1. E−09	
**4**	49711347..49711912	566	AC119751.4	111837	DCUN1D4	2131087	13	18	9. E−16	7. E−18	
**4**	190179314..190179950	637	AC215524.1	4089	ABC7‐42391500H16.3	5896	3	3	2. E−13	3. E−13	
**5**	49661354..49661926	573	CTD−2013 M15.1	3765383	EMB	734265	15	13	2. E−11	1. E−10	
**6**	61322762..61323316	555	RP1‐91 N13.1	81450	MTRNR2L9	251011	1	1	3. E−02	3. E−06	
**7**	59995570..59996144	575	RP11‐548 K12.13	2159318	RP11‐715L17.1	2279216	0	9	no peak	2. E−03	
**8**	43237602..43238166	565	VN1R46P	16792	RP11‐726G23.2	8588	4	4	4. E−08	4. E−09	
**9**	70038064..70038625	562	RN7SL570P	153409	**MAMDC2‐AS1**	0	1	1	8. E−07	1. E−05	MAM Domain Containing 2 (antisense strand)
**10**	42070370..42070986	617	RP11‐96F8.1	3287261	KSR1P1	78323	39	35	9. E−06	2. E−04	
**10**	133688058..133688661	604	AL845259.2	2945	DUX4L29	51944	2	2	2. E−05	3. E−04	
**12**	35614697..35615271	575	AK6P1	1364738	RP11‐125 N22.4	1926740	1	1	8. E−03	5. E−05	
**13**	18211736..18212297	562	AL356585.2	4266	RP11‐341D18.4	41228	1	1	2. E−11	6. E−25	
**16**	34063731..34064293	563	RP11‐598D12.2	12369	CTD−2522B17.8	56176	5	5	7. E−05	2. E−04	
**16**	34587942..34588505	564	BCLAF1P2	319292	CTD−2144E22.9	353471	15	16	8. E−16	7. E−18	
**16**	46394505..46395067	563	PPP1R1AP2	1E+07	ANKRD26P1	74273	16	12	3. E−23	4. E−23	
**17**	21972876..21973349	474	KCNJ18	268263	AC144838.2	83237	9	7	8. E−06	2. E−06	
**17**	26603641..26604283	643	RP11‐846F4.11	3897955	RP11‐260A9.1	377410	17	17	3. E−12	1. E−09	
**18**	107991..108643	653	RP11‐683L23.6	13660	**ROCK1P1**	421	1	2	1. E−07	9. E−10	
**18**	110241..110848	608	**ROCK1P1**	0	MIR8078	1407	2	2	9. E−09	2. E−08	Rho‐Assoc'd Coiled‐Coil Prot. Kinase 1 ψgene1
**20**	31241472..31242039	568	AC104301.2	339650	DEFB115	15624	17	13	3. E−18	5. E−16	
**21**	7926020..7926634	615	SNORA11	99794	CH507‐338C24.3	174616	3	3	9. E−09	4. E−09	
**21**	8806909..8807490	582	SNX18P10	5028	bP−2189O9.1	38075	1	1	2. E−04	1. E−05	
**21**	10269991..10270557	567	CH507‐216 K13.1	132986	AP003900.6	57853	1	4	5. E−03	8. E−06	
**21**	10692593..10693162	570	IGHV1OR21‐1	42757	AP001464.4	2306513	4	2	4. E−15	6. E−15	
**22**	18896073..18896656	584	AC008103.4	11390	DGCR6	9371	1	1	2. E−02	4. E−05	
**X**	156030320..156030900	581	DDX11L16	2442			1	1	1. E−06	1. E−06	
**Y**	10746657..10747217	561	RNA5‐8SP6	546350	RP1‐85D24.4	295504	16	8	5. E−05	1. E−03	
**Y**	11305019..11305652	634	AC134878.1	120061	DUX4L16	1265	6	4	1. E−09	1. E−10	
**Y**	11312236..11312823	588	AC134882.1	3884	DUX4L17	2097	4	5	1. E−08	8. E−09	
**Y**	11324443..11324950	508	DUX4L18	1619	DUX4L19	7378	8	8	3. E−05	4. E−05	
**Y**	11721743..11722304	562	RP11‐295P22.2	247505	RCC2P1	58747	8	8	1. E−09	2. E−08	
**Y**	26638004..26638595	592	FAM58CP	10844	CTBP2P1	3E+07	5	59	8. E−03	3. E−13	

Note

All listed peaks differed significantly from a uniform distribution at *p* < 0.05 to *p *< 6E–57; peak‐coincident genes (at zero distance from peaks) are indicated by bold font. Peak counts were not corrected for the 1.6‐fold higher protein and DNA recovery from AD relative to AMC hippocampus, since all reflect normalized data from 1 µg DNA.

RNA differed from DNA in several important respects. Most notably, 81% of RNA peaks mapped to known or putative genes, vs. only 13% of DNA peaks. (Table 2, Supplementary Tables S2, S3; note that intergenic RNA peaks were omitted to conserve space.) The 49 “within‐peak” genes include 39 (80%) that differed significantly in read count between AD and AMC at *p* < 0.001 (2‐tailed Fisher exact tests), vs. 1 of 38 (2.6%) for DNA. It is noteworthy that 30 significantly differential genes were more abundant in AD, while only 9 (23%) were relatively enriched in AMC. This 3:1 bias is on top of the ~1.8‐fold higher abundance of RNA in AD aggregates (Figure 2A), since all counts were normalized to the source library. Among T98G glioblastoma genes (Supplementary Table S3), 54 of 59 RNA peaks (92%) differ between *APOE3* and *APOE4* at *p* < 0.0001, in marked contrast to DNA peaks of which none were significantly differential.

**TABLE 2:** RNA Fragments Recovered from AD, AMC Aggregates (RNA‐seq analysis)

Chrom.	Region	Length	5’ gene	5’ dist.	3’ gene	3’ dist.	AD reads	AMC reads	AD/AMC reads	Chi^2^ *p*<	Transcript or encoded protein
**1**	633888..634164	277	RP5‐857 K21.3	36389	**RP5‐857 K21.4**	0	515	1360	0.38	0.0001	Transcribed processed pseudogene
**1**	2281959..2282363	405	**SKI**	0	MORN1	38889	1396	1967	0.71	0.0001	Ski proto‐oncogene
**1**	109100211..109100485	275	**SCARNA2**	0	RP5‐1065 J22.4	3049	131	98	1.34	0.05	scaRNA, small Cajal‐body specific RNA2, guides mod'n of snRNAs
**1**	244863775..244864066	292	RP11‐11 N7.5	8277	**HNRNPU**	0	509	653	0.78	0.004	Heterog. Nuclear Ribonucleoprot. U (transripts 1, 3, 4, 8)
**2**	32916221..32916538	318	**LINC00486**	0	AL121656.5	10588	17	5	3.40	0.02	Long Intergenic Non‐Protein Coding RNA 486
**2**	47335292..47335571	280	CALM2	158690	**AC073283.4**	0	2728	2093	1.30	0.0002	Uncharacterized transcript
**2**	148961908..148962183	276	**KIF5C**	0	LYPD6B	75923	3055	1856	1.65	0.0001	Kinesin Family Member 5C (transcript KIF5C_2)
**2**	181678574..181678849	276	ITGA4	142386	**CERKL**	0	107	60	1.78	0.001	Ceramide Kinase Like
**2**	231460607..231460888	282	AC017104.2	7453	**NCL**	0	41	27	1.52	N. S.	Ceroid‐Lipofuscinosis Neuronal Protein 5 (neurodegenerative lysosome storage disease)
**2**	233275721..233275994	274	**ATG16L1**	0	**SCARNA5**	0	400/11	206/5	1.94/2.2	0.0001	Autophagy Related 16‐Like 1 / scaRNA, small Cajal‐body RNA5
**2**	240713854..240714163	310	RP11‐118 M12.2	12168	**KIF1A**	0	2806	4988	0.56	0.0001	Kinesin family member 1A
**3**	160514943..160515216	274	RNU7‐136P	42526	**KPNA4**	0	536	232	2.31	0.0001	Karyopherin (Importin) Subunit Alpha 4
**6**	26204696..26204971	276	**HIST1H4E**	0	HIST1H2BG	11094	164	304	0.54	0.0001	Histone 1 **(AD‐agg‐enriched)**
**6**	34071883..34072162	280	MIR1275	71831	**GRM4**	0	10689	5521	1.94	0.0001	Glutamate Metabotropic Receptor 4
**6**	52995648..52995925	278	**RN7SK**	0	ICK	5353	32569	12138	2.68	0.0001	7SK RNA
**6**	99408034..99408309	276	COQ3	13829	**PNISR**	0	206	135	1.53	0.001	PNN Interact. Ser & Arg‐Rich Prot. (Pinin, desmosome assoc. prot.)
**7**	5529123..5529444	322	MIR589	33205	**ACTB**	0	1210	1719	0.70	0.0001	beta actin **(AD‐agg‐enriched)**
**7**	26200464..26200741	278	CTB−119C2.1	25518	**HNRNPA2B1**	0	168	99	1.70	0.0002	HnRNP A2/B1 **(AD‐agg‐enriched)**
**8**	9903986..9904242	257	MIR597	162217	**LINC00599**	0	1667	1113	1.50	0.0001	Long Intergenic Non‐Protein Coding RNA
**8**	27604977..27605252	276	GULOP	15903	**CLU**	0	156	105	1.49	0.005	Clusterin (a.k.a. ApoJ) **(AD‐agg‐enriched)**
**9**	35657748..35658022	275	SIT1	6797	**RMRP_1**	0	472	184	2.57	0.0001	RNA Component of Mitoch. RNA Processing Endoribonuclease
**10**	101799051..101799385	335	NPM3	15637	**MGEA5 / OGA**	0	683	432	1.58	0.0001	Hyaluronidase =OGA, removes O‐GlcNAc modifications
**11**	35663281..35663611	331	**TRIM44**	0	KRT18P14	196637	1147	592	1.94	0.0001	Tripartite Motif Containing 44
**11**	62841562..62841839	278	RP11‐727F15.9	7518	**WDR74**	0	304	149	2.04	0.0001	WD Repeat Domain 74
**11**	65502716..65503064	349	AP000769.7	4310	**MALAT1_1**	0	30539	16494	1.85	0.0001	Metastasis Assoc. Lung Adenocarcinoma Transcr. 1 (Non‐Protein)
**11**	111911504..111911780	277	RPL37AP8	22029	**CRYAB**	0	228	216	1.06	N. S.	Crystalin A beta, a small HSP **(AD‐agg‐enriched)**
**11**	123061064..123061339	276	RPL31P47	9662	**HSPA8**	0	871	1512	0.58	0.0001	Heat Shock Protein Family A (Hsp70) Member 8 (= HSC70)
**12**	110281619..110281909	291	**ATP2A2**	0	RN7SL769P	78542	433	225	1.92	0.0001	**SERCA2** ATPase Sarcoplasmic/Endopl. Reticulum Ca2+ Transport2
**13**	45975357..45975649	293	AL445232.1	60203	**ZC3H13**	0	372	240	1.55	0.0001	Zinc Finger CCCH‐Type Containing 13
**14**	20343094..20343370	277	SNORD126	16567	**RPPH1**	0	2313	634	3.65	0.0001	Ribonuclease P RNA Component H1
**14**	23321579..23321860	282	**PABPN1**	0	SLC22A17	24445	341	261	1.31	0.01	Poly(A) Binding Protein Nuclear 1
**14**	49586595..49586871	277	RNA5SP384	33846	**RPS29**	0	22800	4958	4.60	0.0001	Ribosomal protein, small subunit 29
**14**	49853648..49853923	276	RNU6‐539P	13820	**Metazoa_SRP_138**	0	24010	7554	3.18	0.0001	RNA, cytoplasmic Signal Recognition Particle
**14**	102084750..102085033	284	RN7SL472P	7277	**HSP90AA1**	0	186	94	1.98	0.0001	HSP 90 family **(AD‐agg‐enriched)**
**17**	6452910..6453209	300	FAM64A	1440	**PITPNM3**	0	671	520	1.29	0.004	PITPNM 3, Phosphatidylinositol Transfer Protein Memb.‐ Assoc.
**17**	19187969..19188246	278	KYNUP1	18300	**SNORD3A**	0	342	99	3.45	0.0001	small nucleolar RNA
**17**	44911190..44911459	270	AC015936.3	6643	**GFAP**	0	7051	8989	0.78	0.0002	Glial Fibrillary Acidic Protein, GFAP (**AD‐agg‐enriched**)
**18**	49814124..49814400	277	ACAA2	163	**RP11‐886H22.1**	0	295	139	2.12	0.0001	Transcribed processed pseudogene
**19**	13298568..13298866	299	CTC−250I14.1	139779	**CACNA1A**	0	10268	5984	1.72	0.0001	Calcium Voltage‐Gated Channel Subunit Alpha1 A
**19**	36304598..36304871	274	**CTD−3162L10.1**	0	LINC00665	8195	556	164	3.39	0.0001	Uncharacterized locus
**19**	44907737..44908015	279	**APOE**	0	CTB−129P6.7	1359	1028	1003	1.02	N. S.	ApoE, Apolipoprotein E **(AD‐agg‐enriched)**
**19**	48645763..48646036	274	DBP	8324	**CA11**	0	407	573	0.71	0.0002	Carbonic Anhydrase 11
**19**	49107777..49108115	339	**SNRNP70**	0	LIN7B	6208	2096	1398	1.50	0.0001	Small Nuclear Ribonucleoprotein U1 Subunit 70
**20**	23637606..23637888	283	CST9	31729	**CST3**	0	573	742	0.77	0.003	Cystatin C, Cystatin 3
**20**	41533110..41533383	274	RP4‐620E11.5	150002	**CHD6**	0	1245	864	1.44	0.0001	Chromodomain Helicase DNA Binding Protein 6, CHDBP6
**21**	8258828..8259116	289	RNA5‐8S5	1894	**FP671120.6**	0	1200	132	9.09	0.0001	Uncharacterized transcript
**21**	8435771..8435981	211	**FP236383.5**	0	FP236383.1	96	207	12	17.25	0.0001	Uncharacterized transcript
**21**	26021821..26022101	281	LLPHP2	258487	**APP**	**0**	1477	702	2.10	0.0001	Amyloid precursor protein, APP, A4 **(AD‐agg‐enriched)**
**X**	140784311..140784609	299	LINC00632	11631	**CDR1**	0	36675	62951	0.58	0.0001	Cerebellar Degeneration Related Protein 1, CDRP1

Note

All peaks shown were significant at *p* < 0.01; peak‐coincident genes (at zero distance from peaks) are indicated by bold font. Read ratios (AD/AMC) and *p* values for AD‐AMC differences were not corrected for 1.6‐fold higher RNA recovery from AD relative to AMC hippocampus, since all normalized RNA‐seq libraries used 1 µg RNA. “AD‐agg enriched” indicates proteins that were also found to be significantly enriched in AD aggregates relative to controls (Ayyadevara, Balasubramaniam, Parcon, et al., 2016).

Aggregate RNA reads that were substantially more abundant in AD than controls (Chi‐square or 2‐tailed Fisher exact *p* < 0.001) include two uncharacterized transcripts on chromosome 21, enriched 17‐fold and 9‐fold beyond other RNAs in AD; ribosomal protein/RPS29, 4.6‐fold; RNAse‐P/RPPH1_2, 3.7‐fold; nucleolar RNA/SNORD3A and long noncoding RNA/LINC00486, enriched 3.5‐ and 3.4‐fold; signal‐recognition‐particle RNAs (SRP_138 and RN7SK), 3.2‐ and 2.7‐fold; mitochondrial RNAse P1/RMRP1, 2.6‐fold; karyopherin/KPNA4, 2.3‐fold; amyloid precursor protein/APP, 2.1‐fold, and SERCA2/ATP2A2, 1.9‐fold (Table 2). It is noteworthy that 2 of the 5 genes identified in aggregate DNA, LINC00486 and RP5‐857K21.4, were also among the AD‐enriched transcripts in aggregates, and 9 of the 39 genes (23%) enriched in AD aggregate RNA, relative to AMC, encode *proteins* that were also enriched in AD‐specific aggregates (Ayyadevara, Balasubramaniam, Parcon, et al., 2016) (bold font in the rightmost column of Table 2).

Because we had observed roughly twice as much DNA in aggregates isolated from glioblastoma cells overexpressing *APOE4*, as in identical cells expressing *APOE3*, we asked whether any particular loci or genes were differentially represented in their aggregates. DNA read counts from E3 and E4 aggregates were in fact quite similar for all DNA loci sequenced (Supplementary Table S1), whereas RNA sequencing data (listed in Supplementary Table S3) show striking increases in aggregate‐entrapped RNA transcripts isolated from *APOE3*‐overexpressing (OE) cells, relative to isogenic cells overexpressing *APOE4*. For 53 of the 59 genes that were confidently identified within fragment alignment peaks, the read count in *APOE4*‐OE cells differed significantly from *APOE3‐OE* cells at Fisher exact *p* < 10^‒4^, with E3/E4 ratios ranging from 1.7‒13.9. Only one gene appeared to be more abundant in the presence of excess *APOE4*, a long noncoding RNA for which there were too few reads to attain significance. This bias is consistent with evidence that APOE4 protein competes with transcription factor TFEB for the ~400 DNA binding sites containing the CLEAR motif, most of which drive expression of proteins involved in autophagy/lysosome functions (Sardiello, 2016).

Differential RNA‐fragment abundances in glioblastoma aggregates, in which only the APOE allele differs between cell lines, tend to be highly significant (53 of 59 have *p* < 0.0001) and comprise an interesting set. Examples include α‐enolase (E3/E4 = 1.85), MHC‐II YBX1 (3.8), scaRNA2 (2.3), histones H2B (2.8) and H1 (6.7), HSP60 (1.6), HSP90‐A1 (1.8) and ‐B1 (1.9), HSP‐A8 (2.0) and HSP‐B1 (3.4), IGF‐BP3 (5.4) and ‐BP5 (2.0), NCL (2.0), prothymosin α (3.2), SPARC (2.4), nucleophosmin (2.5), RACK1 (3.0), 7SK small nuclear RNA (6.2), EEF1‐A1 (2.2), β‐actin (3.2), peptidylprolyl isomerase A (2.6), collagen 1A2 (4.1), vimentin (4.2), CD44 (2.7), cofilin 1 (3.6), GAPDH (2.9), α tubulin (2.9), RNAse P component H1 (9.2), ribosomal proteins RPS2 (4.2) and RPS29 (13.9), 7SL RNA 2 (8.7), β2‐microglobulin (4.1), annexin A2 (3.4), pyruvate kinase M (2.4), profilin 1 (3.7), γ‐actin (3.4), APOE (3.6), ferritin light chain 1 (3.0), galectin 1 (5.0), and filamin A (2.1). With lesser significance, we find synapsin 3 (E3/E4 = 3.5; *p* < 0.0002), sequestosome_1/SQSTM1 (1.3; *p* < 0.004) and vimentin antisense (15; *p* < 0.002).

In both the direction and magnitude of the RNA‐abundance shift, the influence of Alzheimer's disease was less consistent and so appeared less pronounced on average, than that of the *APOE* allele. This is almost certainly due to genetic and environmental variance among AD and AMC subjects (Ayyadevara, Balasubramaniam, Parcon, et al., 2016), in contrast to the single transgene that distinguishes T98G/E3 from T98G/E4 cells. Because all human subjects considered in the present comparison were *APOE3*/*E4* heterozygotes, the AD effect could not have arisen from a difference in *APOE* genotypes. The prevailing reduction in RNA content of E4 aggregates, for the most differentially expressed genes, may reflect transcriptional suppression of TFEB targets by APOE4 (Parcon et al., 2018), rather than an impact of the *APOE* allele on aggregation *per se*.

Mapping the RNA transcripts to the human genome revealed a remarkable cluster of at least 20 intergenic loci in a relatively silent segment (21p11.2‒21p12) of the chromosome 21 short arm (Supplementary Figure S1). While these loci are not differentially represented for the most part, either between AD and AMC or between *APOE3* and *APOE4*, they include 2 loci with the highest AD/AMC ratios we observed, 9.1 and 17.2 (each Chi^2^ *p* < 10^‒6^, Table 2).

### 2.4 Annotation enrichment meta‐analysis of RNA fragments in aggregates

Although we had expected the RNA fragments embedded in aggregates to comprise a random selection from the transcriptome, gene ontology and pathway term enrichment analysis (functional‐annotation clustering in DAVID^TM^, http://david.ncifcrf.gov) revealed highly significant enrichment for specific groups of RNAs. Focusing on genes with RNA reads that map to significant peaks and are differentially abundant in T98G/E3 vs. T98G/E4 glioblastoma cells, DAVID meta‐analysis revealed highly significant enrichment clusters for gene annotations relating to [extracellular exosome + acetylation + phosphoprotein + nucleus, acetylation + poly(A) binding], [Ubl conjugation + cadherin binding + cell‐cell adherens junction ], [glycoprotein binding + protein stabilization], and [myelin sheath + unfolded protein response + protein refolding + stress response + chaperone] (Table 3A).

**TABLE 3:** DAVID Meta‐Analysis of Top RNA‐seq Peaks from Aggregates

A. Genes from E3, E4 reads (55 DAVID IDs, implicating 16 clusters of terms sharing members)				
Cluster #, GO Term	Cluster Enrich.	Count	Fold Enrich.	Benjamini
**1**, Extracellular exosome	10.45	37	5.5	9E−21
Acetylation	10.45	31	4.2	3E−12
Phosphoprotein	10.45	35	2.0	8E−6
Nucleus	10.45	27	2.1	4E−4
**2**, Acetylation	8.65	31	4.2	3E−12
Poly(A) RNA binding	8.65	21	7.1	1E−10
Ubiquitinlike (Ubl) conjugation	8.65	20	5.5	3E−8
**3**, Ubiquitinlike (Ubl) conjugation	6.79	20	5.5	3E−8
Cadherin binding, cell‐cell adhesion	6.79	9	12	6E−5
Cell‐cell adherens junction	6.79	8	12	2E−5
**4**, Glycoprotein binding	3.58	5	30	6E−4
Protein stabilization	3.58	6	17	4E−3
**5**, Myelin sheath	2.77	8	22	2E−6
Response to unfolded protein	2.77	5	45	2E−3
Protein refolding	2.77	4	100	2E−3
Stress response	2.77	5	23	1E−3
Chaperone	2.77	6	14	1E−3

B. Genes from AD, AMC reads (38 DAVID IDs, implicating 9 clusters of terms sharing members)				
Cluster #, GO Term	Cluster Enrich.	Count	Fold Enrich.	Benjamini
**1**. Intracellular ribonucleoprotein complex	3.35	6	26	4E−4
Methylation	3.35	10	6.4	4E−4
Ubiquitinlike (Ubl) conjugation	3.35	10	3.8	2E−2
Poly(A) RNA binding	3.35	9	4.2	5E−2
Acetylation	3.35	12	2.3	8E−2
**2**, Extracellular matrix	2.92	6	12	4E−3
Chaperone	2.92	4	13	4E−2
ATPase activity	2.92	4	12	0.13
**3**, Nucleoplasm	2.82	15	3.2	9E−6
Nucleus	2.82	16	2.0	4E−5
**4**, Myelin sheath	2.17	5	19	5E−3
Chaperone	2.17	4	13	4E−2
Unfolded protein binding	2.17	3	14	0.30

C. Annotations from all RNA reads combined				
GO Term	*p* value	Count	Fold Enrich.	Benjamini
Extracellular exosome	2E−17	42	4.0	3E−15
Extracellular matrix	4E−16	18	16.3	4E−14
Poly(A) RNA binding	4E−13	26	5.7	8E−11
Phosphoprotein	4E−11	55	2.0	2E−09
Acetylation	2E−11	36	3.2	2E−09
Isopeptide bond	3E−11	22	5.9	2E−09
Ubiquitinlike (Ubl) conjugation	4E−11	26	4.6	2E−09
Focal Adhesion	2E−09	14	9.6	1E−07
Myelin sheath	4E−09	10	17.6	2E−07
Membrane	2E−08	27	3.3	1E−06
Identical protein binding	3E−08	17	5.6	3E−06
Intracellular ribonucleoprotein complex	4E−08	9	17.7	1E−06
Disease mutation	4E−08	27	3.2	1E−06
Methylation	1E−07	17	5.1	3E−06
Cytosol	1E−07	32	2.6	4E−06
Glycoprotein binding	2E−07	7	26.7	2E−05
Protein binding	1E−06	55	1.6	6E−05
Oxidation	2E−06	6	56	2E−05
Chaperone	4E−06	8	12	9E−05
Unfolded protein binding	5E−06	7	15.8	2E−04
Neurodegeneration	5E−06	9	9.3	1E−04
Protein stabilization	1E−05	7	13.5	9E−03
Response to unfolded protein	2E−05	5	31.2	7E−03
Cadherin binding, cell‐cell adhesion	2E−05	9	7.7	7E−04
Protein refolding	2E−05	4	70	6E−03
Cell‐cell adherens junction	2E−05	9	7.5	5E−04
MHC class II protein complex binding	3E−05	4	62.1	1E−03
Amyloidosis	1E−04	4	43.2	2E−03
Stress response	3E−04	6	15	5E−03

Note

N.B.: Minor terms were omitted from each cluster. Cluster enrichment is the “Enrichment Score” from Functional Annotation Clustering under DAVID; fold enrichment is “Fold Change” per term; Benjamini indicates the false discovery rate, FDR, predicted by the Benjamini‐Hochberg procedure.

Among genes with well‐mapped reads that differ significantly between aggregates from AD vs. AMC, the most enriched clusters include [intracellular ribonucleoprotein complex + methylation + Ubl conjugation + poly(A) RNA binding + acetylation ], [extracellular matrix + chaperone + ATPase activity], [nucleoplasm + nucleus ], and [myelin sheath + unfolded protein binding + chaperone ] (Table 3B). The very existence of these clusters, and their marked overlap between meta‐analyses derived from gene lists of very different origin (aggregates from cultured glioblastoma cells vs. human hippocampi) despite only 11 common members, suggests that the underlying aggregate‐RNA fragments are strikingly nonrandom in nature. The specific annotation terms that were most enriched (Table 3C) are likely to reflect the nature of proteins that coalesce in AD and AD‐model aggregates, which include terms (fold enrichment) such as protein refolding (70), MHC class II protein complex binding (62), oxidation (56), amyloidosis (43), response to unfolded protein (31), glycoprotein binding (27), intracellular ribonucleoprotein complex (18), unfolded protein binding (16), and neurodegeneration (9).

### 2.5 What mechanisms account for RNA and DNA fragments co‐aggregating with proteins?

What does the inclusion of DNA and RNA fragments imply about aggregates or the mechanism of aggregation? Clearly, there are proteins in aggregates that evolved to bind both nucleic acids and proteins. RNA assumes many transient structures constrained chiefly by its duplex regions, which form A‐helices. DNA, in addition to its repertoire of relatively stable structures (A‐, B‐ and Z‐duplex helices, triplex, and G‐quadruplex forms), in the course of replication and transcription can adopt as wide a range of single‐stranded structures as RNA. Affinity for nucleic acids, as well as the protein constituents of multimeric RNA‐ and DNA‐binding complexes, may require protein structures that are at least partially disordered (Zhang et al., 2013) and/or are highly polar, which in turn may favor aggregation (Babu, 2016; Kovacech et al., 2010). DNA‐binding proteins include histones; high‐mobility‐group (HMG) proteins; constituents of DNA replication, transcription, and repair complexes (e.g. topoisomerases, helicases and polymerases; transcription factors, co‐factors, and repressors); and proteins that stabilize or remodel chromatin (Figure 1) (Li et al., 2006; Mitchell & Tjian, 1989; Stoyanova et al., 2009; Wade, 2001). A key feature shared by many DNA‐binding proteins, in addition to structural instability, is an excess of positively charged residues—allowing formation of electrostatic bonds to the negatively charged phosphates that link DNA‐backbone sugars.

RNA‐binding proteins include splicing factors, translational initiation and elongation factors, ribosomal and associated proteins (e.g., refolding chaperones), signal recognition particles, and proteins involved in the processing and functions of noncoding RNAs (Figure 1) (Castello et al., 2012; Glisovic et al., 2008; Hentze et al., 2018; Turner & Hodson, 2012). There are also diverse proteins that bind both RNA and DNA—including RNA polymerases and other transcription‐complex components, RNA/DNA helicases, and TAR DNA‐binding protein (TDP43/TADBP) (Gao et al., 2019; Hudson & Ortlund, 2014; Kobren & Singh, 2019; Nikpour & Salavati, 2019; Norman et al., 2016; Shi & Berg, 1995; Zacco et al., 2019). Such proteins may account for the presence in aggregates of both RNA and DNA fragments mapping to LINC00486 and RP5‐857K21.4 loci.

G‐quadruplex binding proteins (G4BPs) could be responsible for the presence of certain DNA and RNA segments in aggregates. Of the 39 DNA loci listed in Table 1, 13 (33%) had predicted G‐quadruplex‐forming sequences at >100‐fold higher likelihood than predicted at random, whereas 18 (46%) were <20‐fold above random expectation (Supplementary Table S4 and Figure S2A; note that numbers differ slightly due to binning). This partition into G4‐rich and G4‐poor regions suggests that a subset of DNA fragments may have been “recruited” into aggregates by G4BPs. A similar but less extreme split was observed for RNA fragments listed in this table: 15 of 51 peaks (30%) had ratios >100, vs. 11 (22%) with ratios <20 (Table S2 and Figure S2B).

### 2.6 Viral RNA and DNA fragments are enriched in AD aggregates relative to AMC

RNA and DNA fragments recovered from aggregates include sequences that do not map to the consensus human genome, but are related to known viral sequences compiled in the VirTect Database (Khan et al., 2019; Xia et al., 2019). After removal of all sequence reads homologous to the human genome, the remainder were mapped to the VirTect virus‐sequence library. Raw viral RNA reads comprise 0.09% of total AMC RNA‐fragment sequences (124,803/132,252,624), and 0.15% of AD RNA‐fragments (236,939/158,235,930). Viral DNA reads comprise 0.33% of total AMC or AD DNA‐fragment sequences (124,805/37,225,119 for AMC, 146,271/44,743,221 for AD). In view of their scarcity, such fragments are unlikely to drive aggregation; moreover, only a small minority of total raw reads met all three of the stringent VirTect thresholds (coverage depth ≥5x, a continuous/contiguous region ≥100 nt, and a read count ≥400) required for positive identification of human viruses. A total of 7 human viruses met all criteria, for a total of >135,000 reads (Table 4), out of >800 viruses or viral fragments detected (271,000 DNA reads, 362,000 RNA reads). As a negative control, the *C. elegans* genome was screened with identical parameters, yielding zero viral reads.

**TABLE 4:** Human viral sequences in RNA and DNA fragments recovered from insoluble hippocampal aggregates

Viral Genome Sequence (viral classification group)	AMC_RNAf Reads	AD_RNAf Reads	AMC_DNAf Reads	AD_DNAf Reads	AMC RNA/DNA	AD RNA/DNA	RNA (AD/AMC)	DNA (AD/AMC)
NC_022518.1_HERV_K113 (ssRNA‐RT)	1,289	2,242	3,586	4,698	0.36	0.48	1.74	1.31
NC_001806.1_Human_herpesvirus_1 (dsDNA)	(162)	(327)	192	183	0.84	1.79	2.02	0.95
NC_001798.1_Human_herpesvirus_2 (dsDNA)	12,806	***28,052	1,403	(1,589)	9.13	17.65	2.19	1.13
NC_007605.1_Human_herpesvirus_4 (dsDNA)	(137)	234	200	178	0.69	1.31	1.71	0.89
NC_000898.1_Human_herpesvirus_6B (dsDNA)	(521)	***1,476	2,411	2,627	0.22	0.56	2.83	1.09
NC_012959.1_Human_adenovirus_54 (dsDNA)	273	440	(14)	(10)	19.50	44.00	1.61	0.71
NC_009823.1_Hepatitis_C_virus_type_2 (+ssRNA)	(5,492)	***(15,501)	19,181	(22,028)	0.29	0.70	2.82	1.15
gi|60955|lcl|HPV6REF.1|_Human_papillomavirus_6_(HPV6) (dsDNA)	(82)	(140)	(151)	218	0.54	0.64	1.71	1.44
gi|9627396|lcl|HPV9REF.1|_Human_papillomavirus_9_(HPV9) (dsDNA)	(121)	***(405)	94	(112)	1.29	3.62	3.35	1.19
gi|1491683|lcl|HPV72REF.1|_Human_papillomavirus_72_(HPV72) (dsDNA)	334	**(692)	(22)	(33)	15.18	20.97	2.07	1.50
**TOTALS**	**21,217**	***49,509	**27,254**	**31,676**				

Note

Values in parentheses failed to meet one or more thresholds, but are included here for purposes of comparison. AD RNA counts differ from AMC by Chi‐squared test: **p<0.001; ***p<0.0001.

For 5 of the 10 viruses shown in Table 4, viral RNA fragments were significantly enriched in AD over AMC (at *p* < 0.01 to *p* < 0.0001), relative to the 1.6‐fold AD enrichment of aggregate proteins (Ayyadevara, Balasubramaniam, Parcon, et al., 2016), for a combined significance of *p *< 1E–18. Three of these viruses, all linear duplex DNA viruses, were substantially more abundant in RNA than in DNA: Herpesvirus 2 (RNA/DNA = 9.1 in AMC, 17.7 in AD); Human Adenovirus 54 (RNA/DNA = 19.5 in AMC, 44.0 in AD); and Human Papillomavirus 72 (RNA/DNA = 15.2 in AMC, 21.0 in AD). Most of the remaining viruses were more highly represented in DNA than RNA, signifying that they were transcriptionally inactive. The consistently greater RNA/DNA ratios in AD tissue than in AMC is intrinsically “corrected” for relative viral and aggregate abundance, and strongly implies greater transcription and/or aggregation of RNA in AD hippocampus relative to AMC.

### 2.7 Cotranslational aggregation

As noted above, RNA reads substantially exceeded DNA reads by twofold to fivefold (Figure 2A). The propensity for nucleic acid‐binding proteins to be inherently disordered, suggested above as an explanation for entrapment of nucleic acids in aggregates, is not expected to differ greatly between RNA‐ and DNA‐binding proteins. We propose another mechanism, specific to RNA, that would account for the greater abundance of RNA in aggregates: cotranslational misfolding. Among the RNAs identified in AD‐model aggregates (Table 2), many encode proteins that are themselves enriched in AD aggregates: for example, HnRNP_A2/B1, clusterin/ApoJ, β‐crystallin A (CRYAB), SERCA_2/ATP2A2, GFAP, APOE, and Amyloid Precursor Protein/APP (Ayyadevara, Balasubramaniam, Parcon, et al., 2016). Of the 49 genes with RNA positively identified in aggregates, 9 (18%) encoded proteins that were also identified in aggregates. Twenty‐three (46%) of the same 49 RNAs were significantly more abundant in AD aggregates than in controls, while 6 (12%) were significantly enriched in AD aggregates as both RNA and protein.

During translation, nascent proteins are at high risk for misfolding and aggregation until entire structural domains have emerged from the ribosome. From bacteria to mammals, chaperone complexes that include members of the HSP40, HSP60, and HSP90 families are closely associated with ribosomes, where they counteract misfolding of nascent polypeptides (Deuerling et al., 2019; Zhang & Ignatova, 2011). Nevertheless, the fraction of newly synthesized proteins that is cotranslationally degraded can exceed 50% (Turner & Varshavsky, 2000), indicating that chaperone protection is highly fallible. We wondered whether the remarkable abundance in aggregates of diverse RNA fragments, the great majority of which contain coding sequences, might be a clue that cotranslational aggregation occurs when misfolded, nascent proteins are neither prevented from misfolding nor degraded, prior to their coalescence with other misfolded proteins to form insoluble aggregates.

**FIGURE 3:**
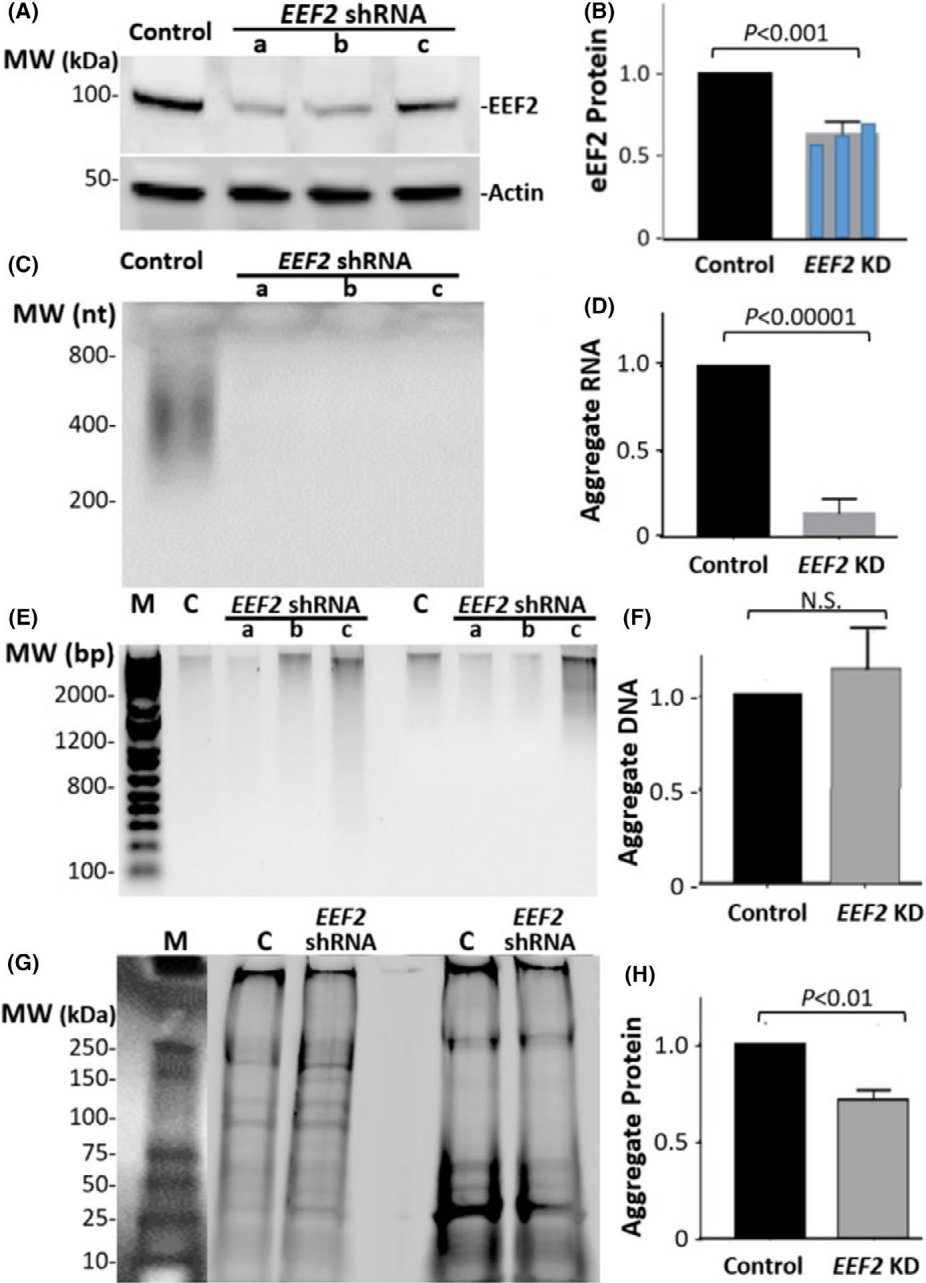
Effects of EEF2 knockdown on the composition of aggregates in SY5Y‐APP_Sw_ cells. Results shown in each panel comprise data from 3 independent cell expansions treated with shRNA constructs targeting EEF2, or 2 scrambled RNAs for controls. Replicate experiments produced similar results. (A, B), Western‐blot quantitation of EEF2 knockdown efficacy, evaluated by EEF2 protein; efficacies of individual shRNAs (constructs a, b, c in Methods) are superimposed in B. (C, D), total RNA fragments in aggregates, quantified by gel staining with SYBR Gold. (E, F) Total DNA fragments in aggregates, quantified by ethidium bromide fluorescence. (G, H) Total aggregate protein, quantified by staining with SYPRO Ruby. *p* values shown here are based on 2‐tailed heteroscedastic t tests, for 3 – 4 experiments, combining data from shRNAs a – c

If this is the case, then interventions that arrest or delay translation should sharply reduce the aggregate content of RNA fragments. We used shRNA knockdown of *EEF2* mRNA, reducing its steady‐state level by 33% (Figure 3A,B) to attenuate protein translation in SY5Y‐APP_Sw_ human neuroblastoma cells. Suppression of *EEF2 *has been shown to extend lifespan, reduce stress response, and improve the balance of protein quality control (Anisimova et al., 2018; David et al., 2010; Tavernarakis, 2008; Turner & Varshavsky, 2000). While prior research showed the existence of co‐translational protein misfolding and degradation (G. Zhang & Ignatova, 2011), our results suggest that slowing translation may reduce aggregation of misfolded proteins, both in *C*. *elegans* (data not shown) and in cultured human cells as follows. In SY5Y‐APP_Sw_ cells, shRNA targeting *EEF2* eliminated over 90% of the RNA entrapped in aggregates (*p* < 0.0001; Figure 3C,D), far exceeding the 33% efficacy of *EEF2* knockdown (Figure 3B). At the same time, this RNAi exposure had little or no effect on aggregate DNA content (Figure 3E,F), but reduced aggregate protein by 30% (*p* < 0.01; Figure 3G,H). In SY5Y‐APP_Sw_ cells treated for 4 h with MG132, a cell‐permeant proteasome inhibitor, aggregates increased 20–30%; however, this rise was not accompanied by any increase in aggregate RNA fragments (Figure S3). This suggests that the reduction in aggregate burden *per se* cannot account for the decline in aggregate RNA after *EEF2* knockdown.

## 3 DISCUSSION

Pathognomonic complexes associated with neurodegenerative diseases, including Alzheimer's, Parkinson's, and Huntington's diseases, are widely termed “protein aggregates” because their diagnostic antigenic markers are proteins. Whether these aggregates also contain other components, however, is a question that has not been adequately addressed. We were aware that some amalgamations of cell debris that accumulate with aging, known as lipofuscin granules, contain a complex mixture of oxidized, glycated and carbonylated proteins, lipids, and possibly other carbohydrates; however, nucleic acids were only rarely noted among their constituents (Cindrova‐Davies et al., 2018; Nowotny et al., 2014). Ginsberg et al. (1998, 1999) reported that 80% of neurofibrillary tangles and 55% of senile (amyloid) plaques can be stained with acridine orange, implying the presence of RNA. Numerous studies have implicated nucleic acid binding by mammalian prion‐like protein, PrP (Cordeiro et al., 2014; Gomes et al., 2012; Macedo et al., 2012; Silva et al., 2008), and the evidence that this extends to other neurodegenerative‐disease seed proteins has been reviewed (Cordeiro et al., 2014).

We were led from the results of proteomic “contactome” studies, intended to define the molecular architecture of aggregates (i.e., which proteins adhere to which other proteins), to investigate the nature, extent, and specificity of nucleic acids incorporated into aggregates. In each of these three respects, the results were unexpected. We observed two‐ to fivefold more RNA than DNA in aggregates, whether isolated from AD or control hippocampus (Figure 2A). Many RNA sequences identified in human hippocampal aggregates were differentially abundant in AD‐ vs. control‐derived aggregates; of these, twice as many were enriched significantly in AD aggregates as in non‐AD controls. Proteomic analyses of aggregates from equal‐weight aliquots of AD vs. AMC hippocampus samples indicate an AD/AMC ratio of 1.84 (*t* test *p* < 0.01), in reasonable agreement with previous AD/AMC protein ratios of 1.65 and 1.66 for Aβ and tau aggregates, respectively (Ayyadevara, Balasubramaniam, Parcon, et al., 2016), and do not differ significantly from the ratios observed here for RNA and DNA. Interactome complexities of Aβ and tau aggregates (unpublished data) indicate AD/AMC ratios of 1.84 and 1.64, respectively (each *t* test *p* < 0.01)—implying more abundant and varied protein interfaces in AD than in AMC hippocampus.

When we compared aggregates isolated from glioblastoma cells overexpressing an *APOE3* vs. *APOE4* transgene, sequences with the most differential representation were quite consistently more abundant in *APOE3*‐bearing cells. We believe this very likely reflects the surprising ability of APOE4 to enter nuclei and bind competitively to the CLEAR/E‐box motifs recognized by transcription factor EB (TFEB), thereby inhibiting expression of autophagy and lysosomal genes (Parcon et al., 2018). Not surprisingly, >90% of aggregate DNA originated from nuclear aggregates, while RNA in aggregates was predominantly of cytoplasmic origin.

Only a small fraction of aggregate‐associated nucleic acids (0.09 – 0.15% of RNA reads, 0.33% of a smaller set of DNA reads) appears to be of viral origin, although these totals may be underestimated due to as‐yet‐uncatalogued and mutated viruses or endogenous retroposons (Sanjuan et al., 2010). The striking 2.3‐fold enrichment of viral RNA sequences in AD aggregates relative to controls, vs. only 1.15‐fold for viral DNA fragments (see Table 4), is consistent with possible roles of viral infection and/or transcriptional activation in the etiology of Alzheimer's disease (Balin & Hudson, 2018; Irish et al., 2009; Kreutz, 2002; Kristensson, 1992; Linet al., 1997; Romeo et al., 2019; Steel & Eslick, 2015). It is also possible that the observed enrichments reflect secondary effects of Alzheimer's pathology, including chronic low‐grade inflammation (Majde, 2010), insofar as they may augment viral infection or transcriptional activation in the AD brain. Previous studies have shown that soluble amyloid‐like proteins bind to nucleic acids, which could lead to formation of amyloid fibrils (Di Domizio et al., 2012). Nucleic acid‐containing amyloid fibrils induce interferon and activate innate immune Toll‐like receptors, driving neuroinflammation and synapse loss in AD (Di Domizio et al., 2012; Roy et al., 2020).

Somatically integrated and even endogenous (heritable) viral genomes have highly variable insertion sites. As a result, viral RNAs and DNAs require identification by searching a database of known virus genomes. Quantitation of viral sequences may thus be underestimated due to the many human viruses as yet unidentified, plus the high viral mutation rates impeding sequence alignment. Nevertheless, viral RNA and DNA comprise very small fractions of the nucleic acids recovered from aggregates. From an evolutionary perspective, however, they may ultimately be responsible for the perseverance in our genomes of proteins with high levels of disorder and high probability of aggregation—provided only that disorder contributes to the ability to bind viral nucleic acids and/or to sequester them in aggregates.

The observed data are consistent with a scenario in which endogenous retroviruses—of which HERV K113 is the youngest and only actively transposing exemplar (Boller et al., 2008)—and integrated genomic copies of retroviruses (e.g., Hepatitis C viruses) and DNA viruses (e.g., Herpes viruses) become activated and transcribed into RNA. Darwinian selection might favor protein variants that are predisposed to misfold, provided that they disable replication and transcription of viral genomes within cells by entrapping them in aggregates. Variants that enhanced survival of a pandemic by even a few percent would undergo strong selective pressure to sweep the population, becoming the predominant or sole alleles (Karlsson et al., 2014).

Predicted G‐quadruplex‐forming sequences in both DNA and RNA, the best known and most abundant class of four‐stranded nucleic acid structures, are also markedly enriched in AD aggregates. Sequences with G‐quadruplex‐forming potential can be recognized by their binding proteins based on singular structural features; they thus often serve as recognition sites for critical proteins with key surveillance or regulatory functions, such as telomere‐binding proteins, viral‐replication proteins, and gene promotor regions (Brazda et al., 2014).

The observation of consistent functional‐annotation terms and clusters, both within each aggregate type and between the two sources of aggregates, confirms that the particular RNA species found in aggregates are not a random sampling from the transcriptome—but it does not explain the basis for their enrichment. We propose two routes by which nucleic acids can be incorporated into aggregates that form either as a result of aging *per se* or due to an age‐dependent pathology such as Alzheimer's disease: (1) “hitchhiker” or “bystander” entrapment of DNA and RNA, when they are bound by proteins that become misfolded and consequently enmeshed in aggregates; and (2) cotranslational misfolding of proteins in the midst of their translation, which might be expected to also ensnare ribosomes and the mRNAs they are translating. The first mechanism is supported by the remarkably high abundance of DNA‐ and RNA‐binding proteins in the aggregate interactome (Figure 1). The second mechanism is most compellingly supported by the decimation (>10‐fold reduction) of aggregate RNA content following shRNA knockdown of the translational procession factor EEF2. We suspect that cotranslational aggregation occurs preferentially in pathways or processes that involve enzymes with multiple partners, and/or several nucleic acid‐binding proteins—thus accounting for the highly significant enrichments observed in aggregated RNA, for genes annotated with specific clusters of descriptive terms. Note that neither of these explanations attributes a primary or causal role to nucleic acids, through which they would “drive” aggregate accrual. Rather, they are collateral casualties due to misfolding of their attached proteins.

Why did *EEF2* knockdown have a far greater effect on RNA content than protein content of aggregates? This is actually the expected result if cotranslational aggregation accounts for only a minor fraction of the protein deposition in aggregates, but is responsible for 90% of their RNA content. Nascent proteins may misfold transiently during translation, but even mature proteins can misfold over time, as a consequence of post‐translational disturbances such as oxidation, phosphorylation, or alkylation, and other temperature‐ or time‐dependent processes that favor misfolding of pre‐existing proteins. Such processes would continue with little prospect of reversal, for all previously‐synthesized proteins—unabated by translational arrest. RNA, however, may only appear in aggregates when it is bound by a misfolded (and hence aggregation‐prone) protein, or when the RNA is in the process of translation into a nascent protein that has a high probability of transient misfolding and aggregation. Our observations imply that cotranslational aggregation is the predominant route, accounting for at least 90% of aggregate RNA.

Our data suggest that proteostasis in SY5Y‐APP_Sw_ cells, which are subjected to chronic ER stress by continual generation of Aβ_1–42_, is normally insufficient to prevent cotranslational aggregation. However, even moderate alleviation of that stress appears to shift the balance back to sustainable translational proteostasis. Translational inhibition has been reported to lower chronic inflammation (Mazumder et al., 2010), which may be a consequence of reduced protein aggregation, augmented by a disproportionate decrease in aggregate RNA.

## 4 CONCLUSIONS

“Protein aggregates” contain nucleic acid constituents that are highly nonrandom in sequence—making it unlikely that they are artifacts, but instead implying that they contain protein‐binding features (including G‐quadruplexes) that might pull them into aggregates. The number and variety of viral sequences found in aggregates suggests that there may be an evolutionary advantage (i.e., antiviral protection) to the synthesis of nucleic acid binding proteins that readily misfold and thus sequester viral genomes in aggregates. Significant enrichment of viral sequences in AD aggregates, relative to controls, is consistent with roles for integrated viruses in AD susceptibility. The preferential enrichment of RNA over DNA in aggregates may implicate a mechanism specific to transcripts: cotranslational aggregation of polysomes during initial misfolding of nascent polypeptides. This process would likely be quite sensitive to the balance between translation rate and chaperone‐mediated refolding capacity. A critical role of cotranslational entrapment is supported by our observation that shRNA knockdown of the translation elongation factor EEF2, although only 25–35% effective, selectively eliminates at least 90% of RNA in aggregates.

## 5 EXPERIMENTAL PROCEDURES

### 5.1 Preparation of cells

Cells were grown in 75‐cm^2^ flasks, with culture medium comprising Dulbecco's Modified Eagles Medium (DMEM) supplemented with 10% (v/v) fetal bovine serum (FBS) at 37°C, grown in an atmosphere of air supplemented with 5% CO_2_. Cells were harvested and washed with phosphate buffered saline and then digested with 0.25% (w/v) trypsin (Thermo Fisher) at 37°C for 4 min or until cells detach from the surface.

### 5.2 *EEF*‐*2* knockdown

For *EEF*‐*2* gene knockdown, RNAi knockdown was performed with 3 distinct *EEF*‐*2* shRNA sequences, targeting human *EEF*‐*2* (SASI_Hs01_00212218 and SASI_Hs_0022218 from Millipore‐Sigma; s4493 from Thermo Fisher), each introduced separately into SH‐SY5Y‐APP_Sw_ cells. Cells were harvested and replated at a density sufficient to achieve ~80% confluence 72 h later. RNAiMax (Thermo Fisher) was used as the transfection reagent, following the manufacturer's protocol. MISSION shRNA universal negative controls (SIC001 and SIC002, Millipore‐Sigma) were transfected by the same protocol, as negative controls for the *EEF*‐*2* knockdowns. Cells were harvested and flash frozen 72 hours after transfection.

### 5.3 Isolation of sarkosyl‐insoluble aggregates

Aggregates were prepared from Alzheimer's Disease (AD) vs. age‐matched control (AMC) hippocampus; T98G/*APOE3* or T98G/*APOE4* human glial cell pellets; or SY5Y‐APP_Sw_ human neuroblastoma cell pellets. Frozen tissues or cells were pulverized with a mortar and pestle (cooled on dry ice) and suspended in lysis buffer containing 20‐mM HEPES pH 7.4, 0.3‐M NaCl, 2‐mM MgCl_2_, 1% NP40 (w/v), supplemented with phosphatase and protease inhibitors (CalBiochem). Tissue suspensions were lysed in a Teflon homogenizer (2 times 10 s, at 0°C) and sonicated (3 times 10 s, at 0°C). Samples were centrifuged 5 min at 600 × *g* to remove debris. Supernatant protein was quantified and each sample (0.6–1.0 mg) was centrifuged 15 min at 13,000 × *g*. Supernatants (soluble protein) were removed, and to each insoluble pellet the same lysis buffer was added plus 1% (v/v) sarkosyl, and mixed well. Samples were centrifuged 20 min at 100,000  × *g*; supernatants and pellets were recovered as “sarkosyl‐soluble aggregates” and “sarkosyl‐insoluble aggregates”, respectively.

### 5.4 Immunoprecipitation of amyloid beta and tau aggregates

AD and AMC hippocampal tissue samples were pulverized as described above. After removal of debris (centrifugation for 5 min at 1400 × *g*), protein was quantified by the Bradford protein assay. Protein was then gently mixed with magnetic beads coated with antibody to either Aβ_1–42_ (ab11132) or tau (ab80579) for immuno‐pulldown (IP); sarkosyl‐insoluble protein was isolated from the antibody‐bound fractions as described previously (Ayyadevara, Balasubramaniam, Parcon, et al., 2016).

### 5.5 Aggregate contactome generation

Insoluble aggregates isolated from SY5Y‐APP_Sw_ cells as above, were cross‐linked following procedures described previously (Balasubramaniam et al., 2019). In brief, purified aggregates were rinsed, cross‐linked with modified click reagents, digested with trypsin, and the linked peptide pairs were affinity purified using streptavidin‐coated beads to capture the biotin‐coupled crosslinking moiety. Cross‐linked peptide pairs were identified from high‐resolution LC/MS‐MS raw data files, using a modified version of Xlink identifier (Balasubramaniam et al., 2019; Du et al., 2011). Xlink identifier outputs were analyzed with the GePhi software package to calculate the degree (number of interacting partners) of each hub. Because high‐molecular‐weight proteins (e.g., titin) have greater potential to interact with other proteins, spectral hits for each hub were normalized, i.e. divided by the length of that hub protein in amino acids. Identified contactome proteins were categorized by degree, as described previously (Balasubramaniam et al., 2019). Proteins with a high normalized degree (number of interacting partners divided by length in amino acids) or classified as hub‐connectors (connecting 2 or more hub proteins that are not otherwise connected) were pursued by further graph modeling; the Cytoscape package (Shannon et al., 2003) was used with default parameters to construct and visualize graphs.

### 5.6 Isolation and quantitation of nucleic acids in aggregates

For sequencing of nucleic acid fragments from isolated aggregates, RNA and DNA were extracted from sarkosyl‐insoluble material isolated from cultured cells, or from AD and AMC hippocampus, using the Qiagen AllPrep kit following manufacturer's instructions and a protocol in which this kit was shown to recover even small nucleic acid fragments (Pena‐Llopis & Brugarolas, 2013).

To quantify DNA and RNA trapped in sarkosyl‐insoluble aggregates, nucleic acids were extracted and assayed by multiple protocols, with consistent results. These consisted of (1) separation of RNA and DNA fragments using a Qiagen AllPrep DNA/RNA extraction kit according to the manufacturer's protocol, with recovery assayed by absorbance at 260 nm; (2) separation of RNA and DNA fragments with the Qiagen kit, and quantitation by ethidium bromide and/or SYBR Gold after resolution by acrylamide gel electrophoresis; (3) selective enzymatic digestion with RNAse‐free DNAse (Thermo Fisher, CA) and assay by 260‐nm absorption (RNA directly; DNA by subtraction), and (4) using TRI Reagent (Molecular Research Ctr., TR118) to isolate RNA, DNA, and protein in a single protocol. Figure 2 data were obtained by method (3) above.

### 5.7 RNA‐seq and ChIP‐seq analyses

All RNA‐seq and ChIP‐seq analyses were performed by the UT Southwestern Genomics Core, analyzed using the CLC Genomics Workbench. We employed ChIP‐seq to evaluate DNA‐fragment specificity; thus, the primary analytic value is the number of significant peaks, with peak validity assessed by an E value relative to a flat distribution (peak absence). RNA‐seq was preceded by peak validation, just as for ChIP‐seq. Subsequently, valid‐peak reads that map uniquely to exons (“unique exon reads”) were summed as our expression metric, and were used to determine differential expression between groups.

The nucleic acid contig assemblies were quite consistent in size, 579 ± 34 (SD) base pairs in length for DNA peaks, and 291 ± 31 (SD) for RNA‐fragment contigs. The efficiency of ChIP‐seq and RNA‐seq fragment cloning protocols, employed prior to sequencing, is quite sensitive to fragment size. Under normal ChIP‐seq protocols, they would be determined by shearing or sonication, size selection by cloning vector, and/or manual size selection. However, in the case of aggregate nucleic acid fragments, other factors may be influential—such as the size, age, and intracellular location of individual aggregates.

### 5.8 Viral sequence analysis

We employed a modified version of VirTect to scan DNA and RNA fragment sequences from human AD and AMC (age‐matched control) hippocampi. VirTect is a pipeline script that calls a sequence of RNA‐seq pattern‐matching routines (Khan et al., 2019). VirTect retrievals of viral matches to aggregate nucleic acid reads, from 3 AD and 3 AMC brain samples, were filtered using the following parameters (https://github.com/WGLab/VirTect/blob/master/README.md): ≥5x coverage depth, a continuous/contiguous region cutoff of ≥75, and a read count ≥50. Several protocol modifications were made for our pipeline: (1) tophat2 was replaced by **hisat2**; (2) code was optimized to use all available threads; (3) the internal threshold number of reads was reduced in exploratory runs for the purpose of obtaining AD/AMC read ratios, but recommended thresholds were maintained to eliminate false positives in the assignment of valid hits; (4) the modified script was rewritten in Bash, with unnecessary subroutine calls deleted to reduce run‐time. The database screened by VirTect comprises complete sequences of 757 viruses, as described (Khan et al. 2019). Target sequences were not restricted to human viruses, in recognition of the high frequency of zoonoses and multiple‐host pathogens.

### 5.9 G‐quadruplex analyses

We employed two programs to screen RNA and DNA sequences for G‐quadruplex‐forming regions: ***G4CatchAll*** (Doluca, 2019) and ***QGRS Mapper*** (Kikin et al., 2006). Both strands were scanned for each DNA‐fragment sequence, but only strands with G4‐forming potential were pursued in subsequent analyses. The following parameters were used for G4CatchAll: G3L (loop limit) was set to 1.3; G2L (allowing 2‐G loops) was set to 1.3; G4H (enables the G4Hunter algorithm for final evaluation). The following parameters were used for QGRS Mapper: Max. Length 30; Min G‐Group 2; Loop size 0 – 36.

### 5.10 Statistical analyses

Inter‐group differences were tested for significance by 2‐tailed Behrens–Fisher heteroscedastic *t* tests, unless otherwise indicated. These conservative tests are appropriate to small‐sample comparisons in which the intra‐group variance is not well estimated. Comparisons of ratios generally employed Yates chi^2^ (chi‐squared) nondirectional tests, substituting 2‐tailed Fisher Exact tests as required to meet numerical constraints. This conservative replacement is stated in the text but is not made explicit (line by line) in tables to conserve space.

## CONFLICTS OF INTEREST

The authors declare no competing or conflicting interests.

## AUTHOR CONTRIBUTIONS

RJSR and SA designed the study; MB and SA performed aggregate cross‐linking studies; MB undertook subsequent data analysis and network modeling; MB and AG performed additional proteomics data analyses and contactome construction; JJ undertook G‐quadruplex and human‐virus searches, and checked all RNA and DNA sequencing data analyses (based on data and initial CLC bio/Qiagen analyses provided by the Genomics Core facility, University of Texas Southwest Medical Center); RA and SA performed *EEF2*‐shRNA knockdown experiments, and RNA and DNA recovery quantitations; RJSR wrote the manuscript with contributions from SA and MB. All authors read and approved the final manuscript.

## Supporting information

Fig S1‐S3

Table S1

## Data Availability

All data generated or analyzed during this study are included in this published article and its supplementary information files. Any reasonable request for additional data will be honored.
