# Supplementary material for: “Protein aggregates” contain RNA and DNA, entrapped by misfolded proteins but largely rescued by slowing translational elongation
Source: Aging Cell. 2021 Mar 31;20(5):e13326. doi: 10.1111/acel.13326 (PMC8135009; doi:10.1111/acel.13326)

Supplementary Figure 1. Mapping of

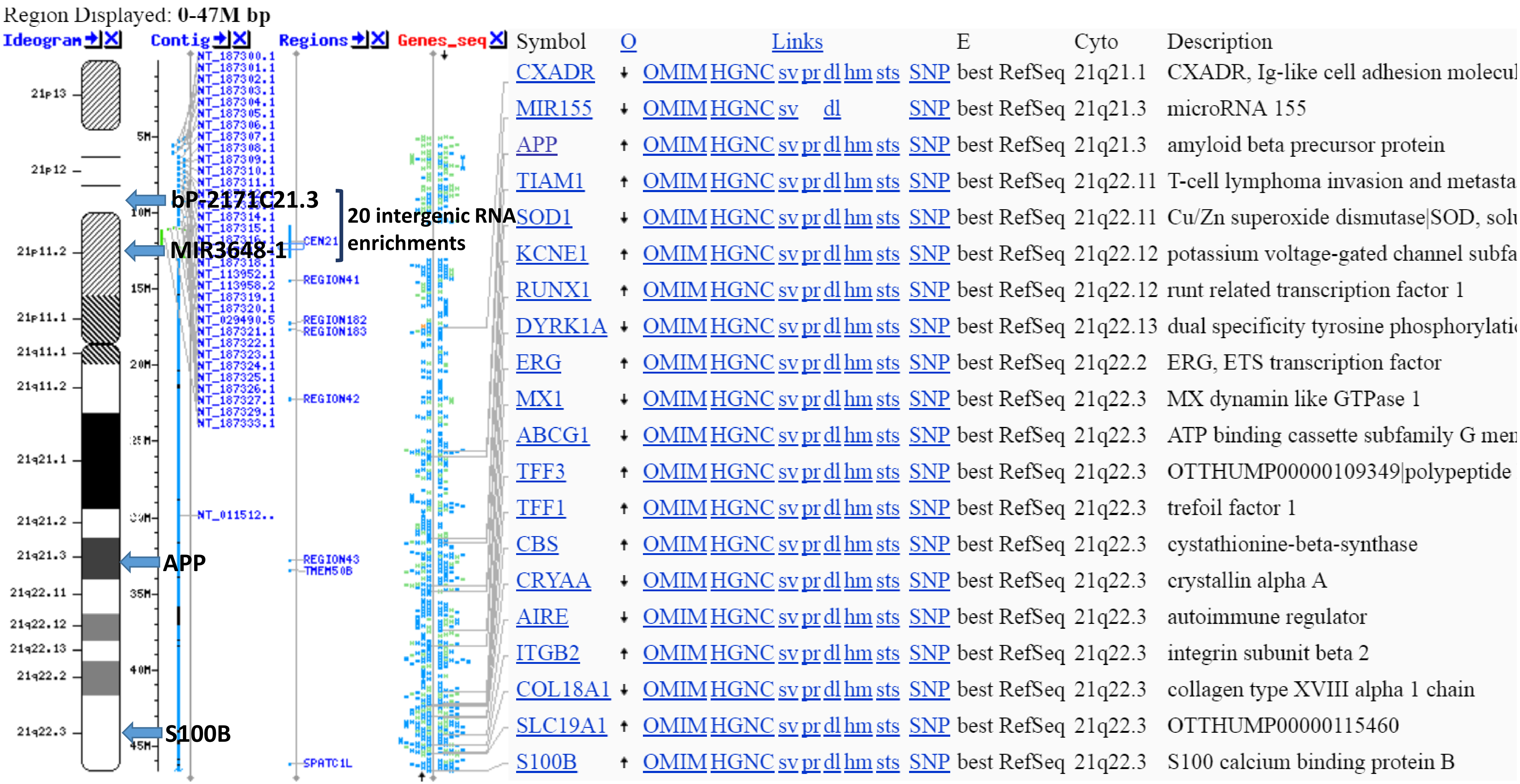

Symbol	O	Links	E	Cyto	Description
CXADR	↓	OMIM HGNC sv pr dl hm sts SNP	best RefSeq	21q21.1	CXADR, Ig-like cell adhesion molecul
MIR155	↓	OMIM HGNC sv dl SNP	best RefSeq	21q21.3	microRNA 155
APP	↑	OMIM HGNC sv pr dl hm sts SNP	best RefSeq	21q21.3	amyloid beta precursor protein
TIAM1	↑	OMIM HGNC sv pr dl hm sts SNP	best RefSeq	21q22.11	T-cell lymphoma invasion and metasta
SOD1	↓	OMIM HGNC sv pr dl hm sts SNP	best RefSeq	21q22.11	Cu/Zn superoxide dismutase|SOD, solu
KCNE1	↑	OMIM HGNC sv pr dl hm sts SNP	best RefSeq	21q22.12	potassium voltage-gated channel subfa
RUNX1	↑	OMIM HGNC sv pr dl hm sts SNP	best RefSeq	21q22.12	runt related transcription factor 1
DYRK1A	↓	OMIM HGNC sv pr dl hm sts SNP	best RefSeq	21q22.13	dual specificity tyrosine phosphorylati
ERG	↑	OMIM HGNC sv pr dl hm sts SNP	best RefSeq	21q22.2	ERG, ETS transcription factor
MX1	↓	OMIM HGNC sv pr dl hm sts SNP	best RefSeq	21q22.3	MX dynamin like GTPase 1
ABCG1	↓	OMIM HGNC sv pr dl hm sts SNP	best RefSeq	21q22.3	ATP binding cassette subfamily G mem
TFF3	↑	OMIM HGNC sv pr dl hm sts SNP	best RefSeq	21q22.3	OTTHUMP00000109349|polypeptide
TFF1	↑	OMIM HGNC sv pr dl hm sts SNP	best RefSeq	21q22.3	trefoil factor 1
CBS	↑	OMIM HGNC sv pr dl hm sts SNP	best RefSeq	21q22.3	cystathionine-beta-synthase
CRYAA	↓	OMIM HGNC sv pr dl hm sts SNP	best RefSeq	21q22.3	crystallin alpha A
AIRE	↓	OMIM HGNC sv pr dl hm sts SNP	best RefSeq	21q22.3	autoimmune regulator
ITGB2	↑	OMIM HGNC sv pr dl hm sts SNP	best RefSeq	21q22.3	integrin subunit beta 2
COL18A1	↓	OMIM HGNC sv pr dl hm sts SNP	best RefSeq	21q22.3	collagen type XVIII alpha 1 chain
SLC19A1	↑	OMIM HGNC sv pr dl hm sts SNP	best RefSeq	21q22.3	OTTHUMP00000115460
S100B	↑	OMIM HGNC sv pr dl hm sts SNP	best RefSeq	21q22.3	S100 calcium binding protein B

Supplementary Figure 2. G-quadruplex forming regions plotted to illustrate the number of quadruplexes per DNA or RNA segment.

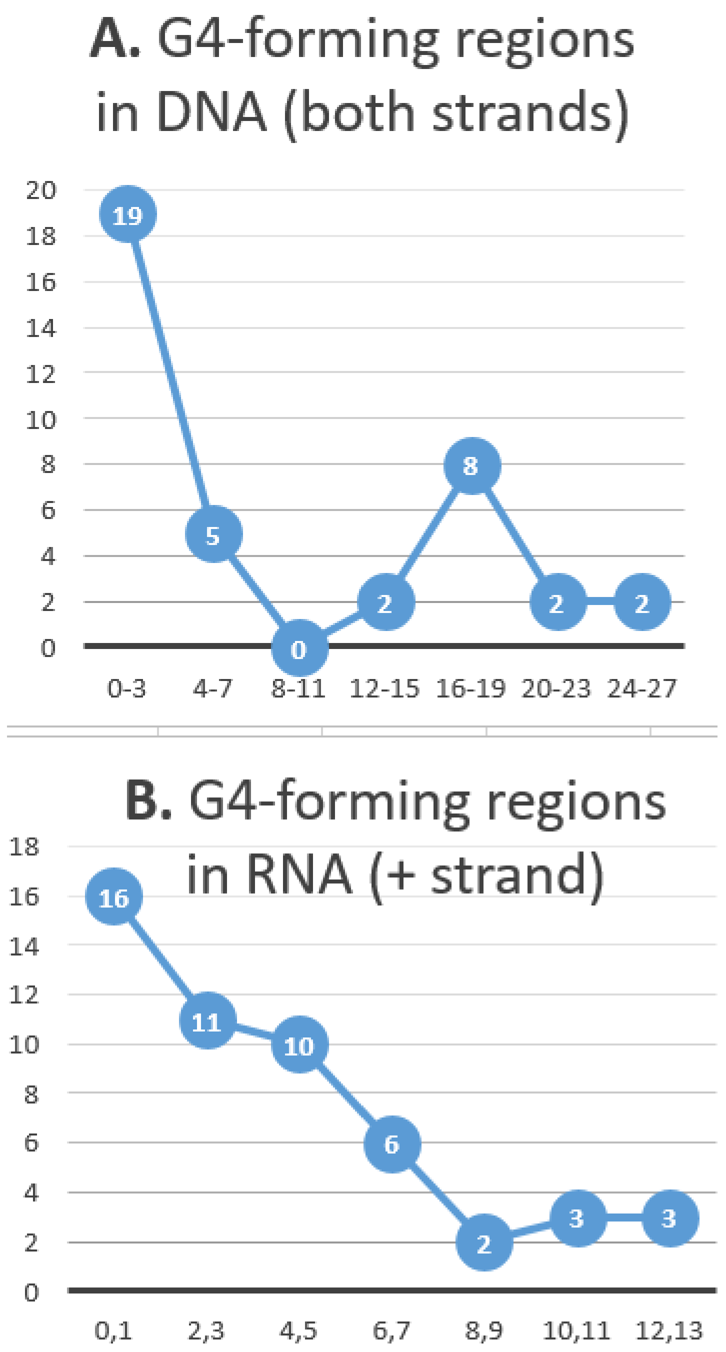

Supplementary Figure 3.

Translational inhibition by *EEF2* knockdown reduces aggregate RNA in SY5Y-APP_{Sw} cells but not significantly in SY5Y cells. Blocking proteasome function with MG132 does not increase the amount of RNA sequestered into aggregates in either cell line.

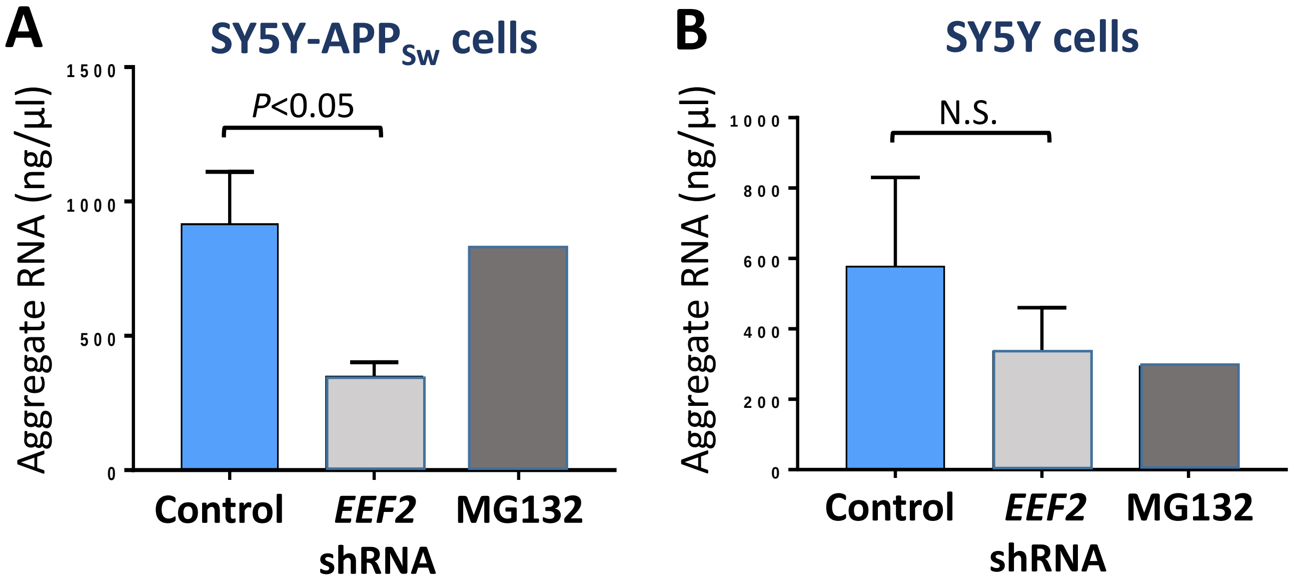

Supplement: Supplementary file 1 — Fig S1‐S3 [file ACEL-20-e13326-s002.pdf]
